# Efficacy and safety of non-pharmacological interventions for gastroesophageal reflux and gastroesophageal reflux disease in children: a systematic review

**DOI:** 10.1186/s13052-026-02264-z

**Published:** 2026-05-07

**Authors:** Giuseppe Banderali, Chiara Mameli, Elena Bozzola, Vita Antonella Di Stefano, Luigi Greco, Carmine Pecoraro, Simone Rugolotto, Elena Scarpato, Sara Sollai, Francesca Vassallo, Elvira Verduci, Massimiliano Orso, Liliana Guadagni, Giovanni Corsello, Rino Agostiniani, Annamaria Staiano

**Affiliations:** 1 Pediatric Unit, Department of Health Science, ASST Santi Paolo e Carlo Hospital, Università di Milano, Milano, Italy; 2 Department of Pediatrics, V Buzzi Children’s Hospital, Università di Milano, Milan, Italy; 3 Department of Biomedical and Clinical Science, Università di Milano, Milan, Italy; 4 Pediatric Unit, Bambino Gesù Children’s Hospital IRCCS, Rome, Italy; 5 Department of Pediatrics, Azienda Ospedaliera per l’Emergenza Cannizzaro, Catania, Italy; 6 General Pediatrician, Heath Care Agency of Bergamo, Bergamo, Italy; 7 Unit of Kidney Transplantation, Nephrology and Dialysis, Santobono Children’s Hospital, Naples, Italy; 8 Division of Pediatrics and Neonatology, Department of Maternal Infant Medicine, Santa Maria della Misericordia Hospital, Rovigo, Italy; 9 Department of Translational Medical Sciences-Section of Pediatrics, University of Naples “Federico II”, Naples, Italy; 10 Department of Pediatrics, Santa Maria Annunziata Hospital, ASL Toscana Centro, Florence, Italy; 11 Department of Health Sciences, University of Milano, Milano, Italy; 12 C. R. E. A. Sanità (Centre for Applied Economic Research in Healthcare), Rome, Italy; 13 Department of Surgical and Biomedical Sciences, University of Perugia, Perugia, Italy; 14 University Department PROMISE “G. D’Alessandro”, University of Palermo, Palermo, Italy; 15 Department of Pediatrics, San Jacopo Hospital, Pistoia, Italy; 16 ASL Toscana Centro, Florence, Italy

**Keywords:** Gastroesophageal reflux, Gastroesophageal reflux disease, GERD, Infants, Children, Non-pharmacological interventions, Dietary therapy, Probiotics, Positioning, Systematic review

## Abstract

**Background:**

Gastroesophageal reflux (GER) and gastroesophageal reflux disease (GERD) are common in infants and children. Non-pharmacological approaches are widely used, but their efficacy and safety remain uncertain. This systematic review evaluates the current evidence on non-pharmacological interventions for pediatric GER and GERD.

**Methods:**

We conducted a systematic review following Cochrane methodology and PRISMA 2020 guidelines (PROSPERO: CRD420251041380). We included randomized controlled trials and systematic reviews of non-pharmacological interventions for GER or GERD in individuals aged 0–18 years. Eligible interventions included dietary modifications, positioning, alginates, probiotics, massage, and complementary therapies. Study selection, data extraction, and risk of bias assessment were performed in duplicate. Due to heterogeneity, meta-analyses were not conducted. Certainty of evidence was assessed using the GRADE approach.

**Results:**

We included 40 studies: 39 RCTs (15 crossover) and one systematic review. Most studies involved infants with uncomplicated GER or GERD. Interventions included dietary modifications (*n* = 25), probiotics (*n* = 3), alginates (*n* = 4), positioning (*n* = 6), and massage therapy (*n* = 2). Most trials reported regurgitation or Infant Gastro-Esophageal Reflux Questionnaire Revised as primary outcomes. Several interventions, especially thickened feeds, probiotics, alginates, and left lateral positioning, were associated with reduced regurgitation frequency. Risk of bias was frequently high, and GRADE certainty ranged from very low to moderate, depending on outcome and intervention type.

**Conclusions:**

Thickened formulas and alginates showed the most consistent symptom improvement in infants with GER or GERD, though overall evidence quality was low to moderate. Other interventions yielded mixed results. Non-pharmacological strategies appear generally safe, but further high-quality research is needed to support clinical decision-making.

**Supplementary Information:**

The online version contains supplementary material available at 10.1186/s13052-026-02264-z.

## Background

Gastroesophageal reflux (GER) and gastroesophageal reflux disease (GERD) are common conditions in the pediatric population, particularly among infants [1]. While GER is often a physiological process, GERD is characterized by more severe and persistent symptoms, that may lead to complications, such as esophagitis, feeding disorders or failure to thrive [2]. Pharmacological treatments, especially proton pump inhibitors (PPIs) and H2-receptor antagonists, are commonly prescribed for pediatric GERD. However, concerns about the safety and long-term effects of these medications in children have prompted increased interest in non-pharmacological interventions [3].

The joint pediatric GER guidelines of the North American Society for Pediatric Gastroenterology, Hepatology, and Nutrition (NASPGHAN) and the European Society for Pediatric Gastroenterology, Hepatology, and Nutrition (ESPGHAN) emphasize the importance of non-pharmacological approaches as first-line treatments for GER and GERD, in infants and children [4]. These strategies aim to reduce symptoms, promote comfort and improve quality of life, minimizing the risks associated with medication use in young children [4].

Feeding modifications represent one of the most used strategies. For formula-fed infants, reducing feeding volumes in overfed infants or offering smaller and more frequent feeds may decrease reflux episodes. The addition of thickening agents (e.g. rice and corn starch) to regular formula, or the use of special thickened formulas (anti-reflux – AR - formulas) aims to reduce the spitting of stomach content by increasing their thickness, though they do not improve acid reflux parameters (e.g. number of acid GER episodes per hour) [5, 6]. However, the addition of starch significantly increases the energy content of the feed, and may lead to overfeeding [7]. Thickening of expressed breast milk is possible, but should be considered only in case of extreme distress of the parents [7]. Positional therapy (e.g., head elevation, left-lateral, and prone positions) could help to reduce episodes of regurgitation, but is currently not recommended for treating GER symptoms in sleeping infants, due to the risk of sudden infant death syndrome (SIDS) [8]. In children and adolescents, lifestyle and behavioral changes, such as avoidance of tobacco/alcohol and trigger foods, are recommended mainly based on adults’ data [9]. In addition, since obesity represents a risk factor for GER symptoms, weight loss in overweight subjects is generally suggested [10]. Moreover, complete and updated parental education and reassurance can optimize GER management, reducing the inappropriate use of medications or dietary interventions [11]. Additional interventions proposed for GER management include dietary supplementations, such as prebiotics and probiotics [12, 13].

Despite their frequent use in clinical practice, the efficacy and safety of non-pharmacological interventions remain inconsistently reported and are often based on low-quality evidence or expert opinion [4]. Therefore, an updated and comprehensive synthesis of existing literature is essential to guide clinicians in making evidence-based decisions for the management of GER and GERD in pediatric populations.

This systematic review aims to evaluate and summarize current evidence on the efficacy and safety of non-pharmacological interventions for GER and GERD in children.

## Materials and methods

This systematic review was conducted in accordance with the Cochrane methodology [14] and reported following the PRISMA 2020 guidelines [15, 16]. The review protocol was registered in the PROSPERO database (CRD420251041380) and was developed to address the following research question: What is the effectiveness of different non-pharmacological treatment options for GER and GERD in infants, children, and adolescents?

The inclusion criteria adhering to the PICO(S) framework (Population, Intervention, Comparator, Outcome, Study Design) are reported in Table 1.

**Table 1:** Inclusion criteria based on the PICO(S) framework

Population	Infants, children, and adolescents aged 0–18 years with a confirmed diagnosis of GER or GERD based on any recognized clinical or diagnostic criteria.
Intervention	Non-pharmacological strategies, such as dietary modifications, feeding interventions, positional therapy, behavioral therapy, alginates, massage therapy, and other complementary therapies.
Comparator	Other non-pharmacological treatments, placebo, or no treatment
Outcomes	• Frequency of vomiting/regurgitation, • Score on the Infant Gastro-Esophageal Reflux Questionnaire Revised (I-GERQ-R) • Total number of reflux events • Estimated volume regurgitated • Frequency of respiratory symptoms (including nocturnal cough and asthma) • Weight gain, and • Adverse events
Study design	Systematic reviews/meta-analyses of randomized controlled trials (RCTs) and individual RCTs.

Systematic literature searches were performed in PubMed, Embase, and Web of Science. Search strings were built using both MeSH terms and free-text keywords aligned with the PICO framework. The selection process was performed by two independent reviewers (MO and LG).

Full search strategies and a detailed description of the methods are provided in Additional File 1. Excluded studies and reasons for exclusion are listed in Additional File 2.

The risk of bias assessment was performed using the Cochrane RoB 2 tool for individual RCTs [17], while systematic reviews were assessed using the JBI Critical Appraisal Checklist for Systematic Reviews and Research Syntheses [18].

Meta-analyses were not performed because of the limited number of studies available for each comparison and heterogeneity in PICO elements. Results were therefore presented using summary tables and GRADE evidence profiles. Continuous outcomes were summarized using means with standard deviations/standard errors, or medians with interquartile ranges; categorical outcomes were presented as frequencies and percentages. Reported p-values were retained from source studies, with *p* < 0.05 treated as statistically significant.

Due to the small number of studies per comparison, publication bias was not assessed. Certainty assessment for critical outcomes was graded using GRADE (high/moderate/low/very low) approach [19], and evidence profiles were generated with GRADEpro GDT software [20].

## Results

**Fig. 1:**
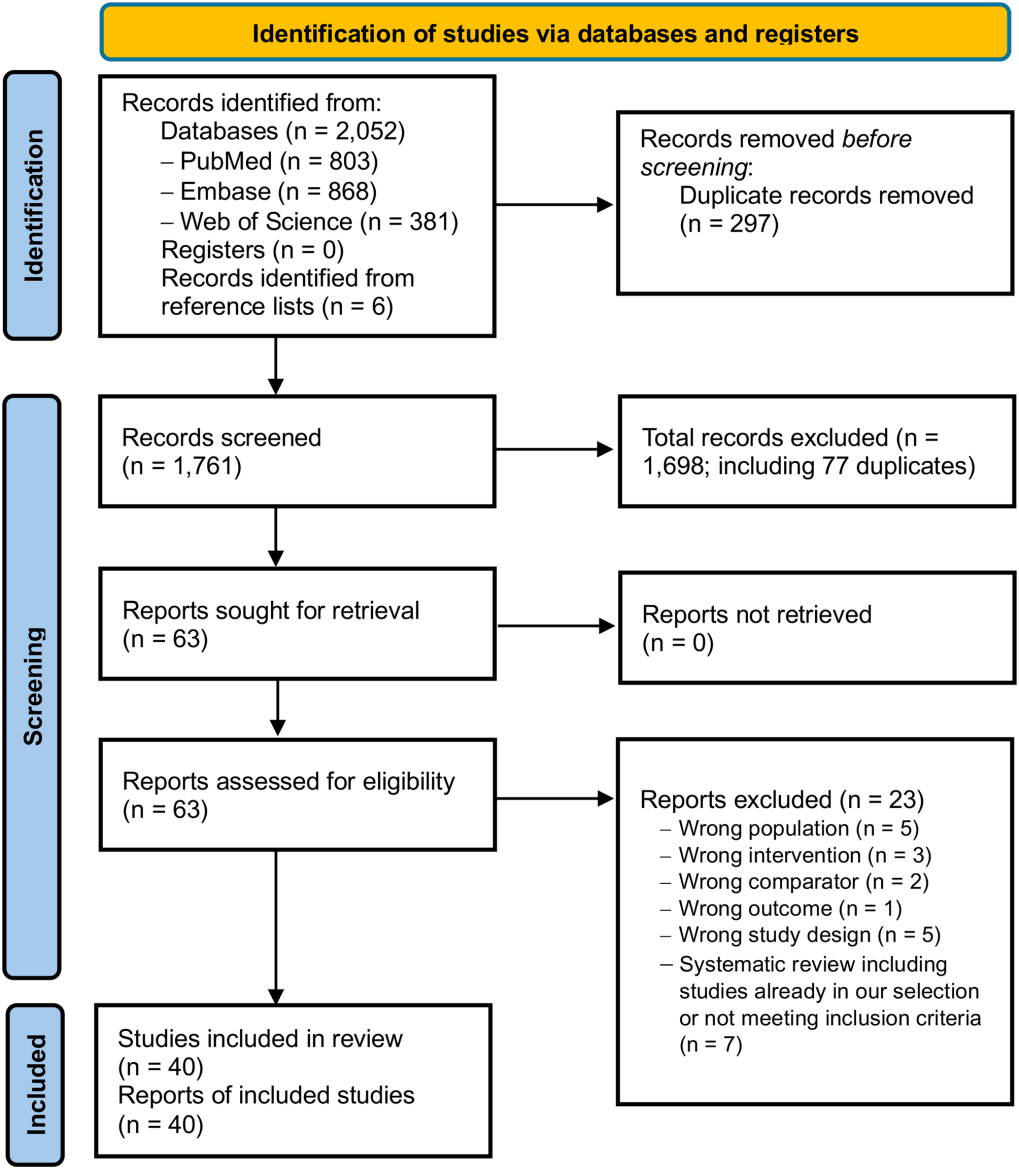
PRISMA 2020 flow diagram

A total of 2,052 records were identified through the systematic literature search: 803 from PubMed, 868 from Embase, and 381 from Web of Science. In addition, six further records were identified by screening the reference lists of relevant articles. After removing duplicates, 1,761 records remained for title and abstract screening. Of these, 63 full-text articles were assessed for eligibility, resulting in the inclusion of 40 studies [5, 8, 21–58] in the final review (Fig. 1). A list of excluded studies along with reasons for exclusion is provided in Additional File 2.

### Study characteristics

The main characteristics of included studies are shown in Additional File 3. The included studies were published between 1983 and 2024. The majority of clinical trials (32 out of 39) were conducted in a single country, while 7 trials were multinational. The most frequently represented countries were Italy (11 studies), Belgium (8), the United States (7), and France, Japan and United Kingdom (3 each).

Of the 40 included studies, 39 were RCTs, of which 15 employed a crossover design. One study [28] was a systematic review of RCTs. The included studies primarily involved infants and, to a lesser extent, preterm or very low birth weight infants. Most studies focused on otherwise healthy infants with frequent regurgitation or uncomplicated GER. A smaller number of studies specifically included infants with GERD [21, 31, 33, 39], often diagnosed based on clinical criteria or pH monitoring. Participant ages ranged from birth to 12 months, with many studies enrolling infants between 0 and 6 months. Only one study [56] included children up to 12 years old. A few studies focused specifically on preterm infants or those with very low birth weight [8, 25–27].

The sample sizes of the 39 RCTs ranged from 5 to 960 participants, with a median of 53 infants and an interquartile range of 70. Seventeen studies (43.6%) enrolled fewer than 50 infants, 14 (35.9%) enrolled between 50 and 100, and 8 (20.5%) enrolled more than 100 infants.

The included studies assessed a wide range of non-pharmacological interventions for managing GER or GERD in infants. The majority of trials (*n* = 25) evaluated dietary modifications. Probiotics were investigated in three studies [21, 28, 32]. Six studies [8, 34, 40–42, 46] assessed positioning therapy. Four trials [55–58] evaluated alginates or alginate-based reflux suppressants. Two studies [33, 39] examined manual therapies.

The comparators used in the included studies were primarily standard formulas or alternative non-pharmacological dietary interventions, such as different thickening agents or protein compositions. Several studies used placebo or no treatment as the control, particularly in trials evaluating probiotics and alginates. In trials investigating positioning therapy, comparators consisted of alternative body positions (e.g., prone vs. supine or lateral). A few studies comparing manual therapy interventions used sham massage or massage without active ingredients.

### Risk of bias assessment

Risk of bias was assessed using the Cochrane RoB 2 tool across five domains. When grouped by intervention type, notable differences in the risk profiles emerged:

- *Dietary modifications*: These studies frequently showed high risk of bias in Domain 4 (bias in outcome measurement), largely due to unblinded outcome assessment of subjective endpoints like regurgitation frequency or symptom scores. Several studies also showed some concerns in Domain 2 (deviations from intended interventions), especially when parental behavior could influence feeding practices.
- *Probiotics*: Most probiotic trials presented some concerns in Domain 1 (randomization process), but generally had low risk in other domains, particularly when placebo controls were used and objective outcomes (e.g., weight gain) were assessed.
- *Positioning therapy*: These studies commonly exhibited high risk in Domain 4, again due to lack of blinding and use of subjective outcomes (e.g., caregiver-reported symptoms). Some also showed some concerns in Domain 2, also due to lack of blinding.
- *Alginates*: Three studies used placebo as comparator and generally achieved low risk in Domains 3 and 5 but often had some concerns or high risk in Domains 1 and 4 due to limited reporting on allocation concealment and subjective outcome measurement.
- *Manual therapy*: These studies had mixed risk profiles, with one trial rated as low risk across domains, and another showing high risk in Domains 4 and 5, largely due to lack of blinding and selective reporting.

Overall, bias in outcome measurement (Domain 4) was the most frequent concern across intervention types, particularly in studies relying on parental reporting without blinding. Studies that included objective measures, such as pH monitoring or weight gain, more often achieved low risk ratings.

Full risk of bias assessments for each study and outcome are provided in Additional File 4.

### Summary of results by intervention type

#### Dietary interventions: thickened formula, hydrolyzed formula and fortified milk

The search identified 17 studies on thickened feedings [5, 22–24, 29, 30, 35–38, 44, 45, 47, 50, 52–54], one on soy-based formula with added soy fiber [43], two on extensively hydrolyzed protein-based formulas [26, 48], three on partially hydrolyzed protein-based formulas [31, 49, 51] and two on maternal milk fortifiers [25, 27].

GRADE assessment indicated very low certainty of evidence for most RCTs [23–25, 29, 35–38, 49], mostly due to very serious risk of bias and small sample size. Four studies were rated as low quality [26, 44, 47, 48], and three as moderate [22, 53, 54]. Ostrom 2006 [43] had a moderate level of certainty for efficacy outcomes (vomiting/regurgitation frequency and weight gain) and low for safety outcomes (report of adverse events). Two other RCTs [51, 52] were assessed low for safety outcomes, while were deemed high for efficacy outcomes (vomiting/regurgitation frequency, esteemed volume regurgitated and weight gain). One study reached high certainty for efficacy outcomes (vomiting/regurgitation frequency, IGSQ score, and estimated volume regurgitated) and moderate for safety outcomes [45].

Eleven RCTs have compared various thickened formulas with standard formula [23, 29, 30, 35–38, 50, 52–54], two compared them with rice flour thickened formulas [5, 44], two with different formulas enriched with postbiotics [22, 45], one with a partially hydrolyzed protein-based thickened formula [51], one with parental counseling and lifestyle modifications [47], and one with infant positioning [24].

The study populations of most RCTs consisted of infants with uncomplicated GER or GERD. Three studies focused on preterm infants [8, 25, 26], one on very low birth weight and very preterm infants [27], and one on infants with suspected cow’s milk allergy [48]. In addition, one study [56] included children up to 12 years old.

Most studies have evaluated clinical benefits by measuring reductions in the frequency of regurgitation, vomiting episodes, and GER clinical scores. In a few trials, the volume of GER has also been assessed [36, 37, 45, 52]. Six studies considered symptoms alongside esophageal pH and/or pH-impedance monitoring [5, 38, 44, 50, 53, 54]. In three studies [23, 35, 37], gastric emptying was assessed through scintigraphy or ultrasonographic emptying studies.

Vomiting/regurgitation frequency was the primary efficacy outcome in most trials [23, 24, 29, 36, 37, 43–45, 47, 51–54]. Three studies [5, 45, 47] used the I-GERQ-R to evaluate symptoms.

Ummarino et al. [47] compared the efficacy of a rice starch thickened formula (reported as 14.3 g/100 ml) to a standard formula and to treatment with magnesium alginate plus simethicone. All three groups showed a significant reduction in regurgitation after two months of intervention compared to baseline (*p* < 0.038, *p* < 0.03, and *p* < 0.002 respectively).

One study [52] noted improvements in choking, gagging, and coughing episodes as well as sleep quality in the thickened feed group, from baseline to week 5 of treatment (*p* = 0.049 and *p* = 0.030, respectively).

The main thickening agents used were locust bean gum, cornstarch, and rice starch.

Ostrom (2006) [43] assessed the effect of a soy-fiber-thickened formula showing a significant reduction in regurgitation episodes after four weeks of intervention, compared to standard formula (*p* = 0.029).

Vandenplas 2014 [48] treated regurgitating infants with suspected cow’s milk allergy with both thickened and non-thickened casein extensively hydrolyzed formulas that significantly reduced regurgitation in all infants, compared to baseline (*p* < 0.001).

In another RCT, Vandenplas 2008 [49] treated infants with persistent regurgitation, resulting in a significant decrease of regurgitation (*p* = 0.002) and crying (*p* = 0.003), with a double thickened (bean gum and processed tapioca starch) whey-based partially hydrolyzed formula, compared to a single-thickened casein-predominant formula.

In another double-blind, crossover RCT [51], there was a reduction in regurgitation frequency with two thickened formulas, with statistically better results for the partially hydrolyzed whey-based formula (*p* < 0.0001).

Another double-blind RCT by Indrio et al. [31] evaluated the impact of a formula with partially hydrolyzed whey protein, starch, and *Lactobacillus reuteri* (DSM 17938) on gastric emptying and regurgitation. The treatment reduced the number of daily regurgitations in the intervention group, compared to the control group treated with a 70% whey protein and 30% casein formula (*p* < 0.0001).

Our research has identified two studies on maternal milk fortification [25, 27] and its effects on GER. The first study [25] assessed the impact of fortified human milk thickened with precooked starch or non-thickened in preterm infants and found no significant difference between the two groups (*p* > 0.05).

The second study [27] compared the effects of a human milk fortifier derived from donkey milk with those of a bovine milk fortifier, in very low birth weight infants, noticing a reduction in GER episodes evaluated with multichannel intraluminal impedance (MII) in the donkey milk group (*p* = 0.036).

The majority of RCTs showed a statistically significant reduction of symptoms between the intervention group with thickened feed compared to controls [5, 23, 24, 26, 27, 37, 38, 43–45, 49, 51–54]. Miyazawa 2007 [35] found a statistically significant reduction in regurgitation frequency in infants fed with thickened formula compared to controls (*p* = 0.00048), but no difference in weight gain, gastric emptying rate and feeding time. Other studies reported a statistically significant reduction in efficacy outcomes before and after treatment, but no difference between intervention and control groups [22, 25, 29, 30, 36, 47, 48, 50].

#### Probiotics

We identified three studies on probiotics: two RCTs [21, 32], and one systematic review [28]. In line with our inclusion criteria, we excluded studies that investigated probiotics for prophylactic purposes and included only those where probiotics were used as an intervention in children already diagnosed with GER or GERD. The included meta-analysis partially addressed our PICO question, as it combined both prophylactic and therapeutic studies. Another study [31] comparing a thickened formula enriched with probiotics to a standard formula without probiotics, was described in the previous paragraph.

In the randomized controlled trial conducted by Indrio et al. (2011) [32], the administration of *Lactobacillus reuteri* DSM 17,938 for 28 days significantly reduced the frequency of regurgitation in infants with functional gastroesophageal reflux. At the end of the treatment period, the median number of regurgitation episodes per day was 1.0 (range 1.0–2.0) in the probiotic group compared to 4.0 (range 3.0–5.0) in the placebo group (*P* < 0.001; GRADE certainty: low). This clinically relevant improvement was also accompanied by a significant acceleration of gastric emptying and a reduction in fasting antral area.

In a large multicenter RCT, Baldassarre et al. [21] evaluated the effect of *Bifidobacterium animalis* subsp. *lactis* BB-12 (six daily drops = 1 × 10⁹ colony-forming units [CFU]) in 960 formula-fed infants with persistent regurgitation. After two months of treatment, the BB-12 group showed a significantly greater reduction in the proportion of infants with a positive clinical GER score (I-GERQ-R) compared to the control group, which received no supplementation (from 25.8% to 14.7% vs. from 31.7% to 50.7%). Mean total I-GERQ-R scores also decreased significantly in the BB-12 group, from 22.3 at baseline to 11.0 at two months, whereas scores in the control group remained consistently high (23.0 to 21.0; *p* < 0.001; GRADE certainty: low).

#### Alginates

Our research identified three trials [55–57] on alginates and one on alginate-based reflux suppressants [58]. Two studies compared alginates vs. placebo. The study by Buts et al. (1987) [56] assessed clinical symptoms and esophageal pH monitoring in a small cohort of infants and children (10 in the alginate group and 10 in the placebo group), most of whom had non-erosive esophagitis. The alginate group showed significant reductions across all reflux parameters, including the total number of reflux episodes, percentage of reflux time, and mean duration of reflux episodes (all *p* < 0.05), with improvements ranging from 35% to 61% compared to baseline. In contrast, no significant changes were observed in the placebo group. No adverse effects were reported in either group (GRADE certainty: very low).

Another small crossover RCT by Del Buono et al. (2005) [57] evaluated the effect of sodium and magnesium alginate and mannitol compared to placebo on GER in 20 bottle-fed infants with clinically suspected GER. Over 24 h, a total of 747 reflux episodes were recorded, with no significant differences between treatment arms in the number of total reflux events, acid reflux episodes, acid clearance time, or reflux duration. The only statistically significant difference observed was a modest reduction in the average height of refluxate migration within the esophagus after administration (median 66.6% of esophageal length), compared to placebo (77.3%, *p* < 0.001). No adverse effects were reported.

Miller (1999) [58] conducted a multicenter, double-blind, RCT comparing an aluminium-free pediatric alginate formulation with placebo, in 88 infants (42 alginate, 46 placebo) with recurrent GER. After 14 days of treatment, the alginate group showed a significant reduction in vomiting/regurgitation episodes compared to placebo (median reduction from 8.5 to 3.0 vs. from 7.0 to 5.0; *p* = 0.009; GRADE certainty: low). A non-significant trend towards reduced severity of vomiting was also observed (*p* = 0.061). No significant differences were reported in the incidence or type of adverse events between groups.

A more recent crossover RCT conducted by Baldassarre et al. (2019) [55] evaluated the efficacy of a magnesium-alginate formulation in reducing GER symptoms in infants, as measured by the I-GERQ-R. Formula-fed infants (*n* = 53) were randomized to receive either two weeks of magnesium alginate followed by two weeks of thickened formula, or vice versa. Both treatments showed similar efficacy: mean I-GERQ-R score reductions were − 8.96 with alginate and − 9.74 with thickened formula (*p* = 0.48; GRADE certainty: very low).

#### Positioning therapy

Six RCTs [8, 34, 40–42, 46] were included in the review, which reported data on several positions in infants, including prone, left lateral, and head-elevated positions. Another study by Chao and Vandenplas (2007) [24] comparing the effect of cereal-thickened formula vs. postural therapy has been described in the paragraph on thickened formulas.

Ewer et al. (1999) [8] conducted a randomized crossover study in 18 preterm infants with clinically significant GER, using 24-hour esophageal pH monitoring to compare the effects of three positions: prone, left lateral, and right lateral. Each infant was placed in each position for 8 h. The prone and left lateral positions significantly reduced all reflux parameters compared to the right lateral position, including reflux index, number of episodes, and duration of the longest episode (all *p* < 0.001; GRADE certainty: very low). No adverse effects were reported.

Orenstein et al. (1983) [41] conducted a crossover RCT involving 15 infants under 6 months of age with confirmed GER. The study compared prone, head-elevated positioning in a cloth harness to a 60-degree upright infant seat, using two-hour postprandial pH monitoring sessions. Across 19 paired trials, the harness position significantly reduced all reflux parameters, including percent time with pH < 4 (7.9% vs. 37.4%), number of episodes (5.2 vs. 19.6), and episode duration (all *p* < 0.05; GRADE certainty: very low). The prone-elevated harness was superior to the upright seat in reducing GER, with no adverse effects reported.

In the same year, Orenstein et al. (1983) [42] conducted another crossover RCT in nine infants under six months of age with documented GER, comparing positioning in an upright infant seat (60°) versus the prone horizontal position, using two-hour postprandial esophageal pH monitoring. Reflux parameters were significantly worse in the seat than in the prone position: the percentage of time with pH < 4 was higher (28.2% vs. 12.8%, *p* = 0.023), as were the number of reflux episodes (16.0 vs. 10.1, *p* = 0.002; GRADE certainty: very low). The authors concluded that the infant seat is detrimental to reflux control compared to simple prone positioning. No adverse events were reported.

Orenstein et al. (1990) [40] conducted a large crossover RCT in 100 infants under 6 months of age with suspected GER, comparing flat prone versus head-elevated prone positioning using esophageal pH monitoring. Among the 90 infants with confirmed abnormal reflux, no significant differences were found between the two positions across 10 reflux parameters. In the overall group, only two parameters improved modestly with head elevation (postprandial frequency of reflux episodes and number of episodes > 5 min), but total reflux time did not differ. The authors concluded that flat prone positioning is as effective as head-elevated prone positioning and preferred due to its simplicity. No adverse effects were reported.

Tobin et al. (1997) [46] conducted a randomized crossover study in 24 infants under 5 months with symptomatic GER (reflux index > 5%), using 48-hour esophageal pH monitoring to compare four positions (supine, prone, left lateral, right lateral), each tested both horizontally and with 30°head elevation. Reflux index was significantly lower in the prone (6.7%) and left lateral (7.7%) positions compared to the supine (15.3%) and right lateral (12.0%) positions (*p* < 0.001). Additionally, the number of reflux episodes normalized for time was significantly reduced in the prone (4.3) position compared to supine (7.1) (*p* = 0.007; GRADE certainty: very low). Head elevation did not significantly affect reflux parameters. The authors concluded that left lateral positioning is a safe and effective alternative to prone for reducing acid reflux in infants.

Loots et al. (2014) [34] conducted a randomized, sham-controlled trial in 51 infants under 6 months with symptoms suggestive of GERD and a positive GER–symptom association. Infants were assigned to four groups combining either proton pump inhibitor (PPI) or antacid (AA) with either left lateral positioning (LLP) or head-of-cot elevation (HE). Considering only the two groups taking antacids + positioning, after two weeks, the AA + LLP group showed a significative reduction in total GER episodes (from 47 to 29; *p* < 0.05; GRADE certainty: moderate). Vomiting significantly improved in the AA + LLP group (from 7 to 2 episodes; *p* < 0.05; GRADE certainty: moderate), while no differences in cough episodes were found. No safety concerns were reported.

#### Massage therapy

The search identified two RCTs [33, 39] on the effects of massage therapy on infants with GERD. Neu et al. (2014) [39] conducted a randomized controlled pilot trial involving 36 infants with GERD symptoms, comparing massage therapy to a non-massage sham treatment, over a 6-week period. GERD symptoms were assessed using I-GERQ-R. Mean I-GERQ-R scores in the massage group decreased from 22.0 (SD 4) at baseline to 14.4 (SD 5) at 6 weeks; similar improvements were seen in the control group (from 23.5 (SD 4) to 13.7 (SD 6)). However, no significant difference between groups was found in ANCOVA analysis. Weight gain increased over time in both groups, with no significant group difference.

Kenari et al. (2020) [33] conducted a randomized controlled trial in 90 infants aged 1–12 months diagnosed with GERD, comparing abdominal massage with mastic gum oil versus massage without oil. Both groups received omeprazole (20 mg every 12 h) for 2 weeks. The primary outcomes included regurgitation, irritability, and other GERD-related symptoms, assessed with the GSQ-I. While both groups showed a significant reduction in total and individual symptom scores over time, no statistically significant difference was observed between the two groups. Specifically, regurgitation scores decreased from 10.8 to 4.3 in the oil group and from 9.3 to 6.5 in the control group (GRADE certainty: very low).

GRADE Evidence Profile tables are available in Additional File 5.

## Discussion

This systematic review evaluated the efficacy and safety of non-pharmacological interventions for the management of GER and GERD in infants and children. Across the 40 included studies − 39 of which were RCTs—a broad range of interventions were assessed, including dietary modifications, positioning therapy, probiotics, alginates, and manual therapies. Most studies focused on otherwise healthy term infants, predominantly under six months of age, while only a few trials addressed preterm or very low birth weight infants [8, 25–27], and only one trial included older children, up to 12 years of age [56].

### Dietary modifications

Thickened formulas represented the most frequently studied dietary modification. The findings from the included trials [5, 7, 22, 24, 29, 30, 35–38, 44, 45, 47, 50, 52–54] consistently indicated a reduction in frequency of vomiting/regurgitation within the intervention groups, suggesting that thickened formulas may contribute to a decrease in overt regurgitation episodes. In addition, 6 studies compared symptoms to esophageal pH and/or pH-impedance monitoring [5, 38, 44, 50, 53, 54], highlighting a positive impact of thickened formulas also on esophageal parameters, with the exception of Wenzl et al. [53] that found no significant difference in mean GER duration/number of acid reflux episodes, and maximum refluxate height in the esophagus between infants administered a carob bean gum-thickened formula or a standard formula. Nevertheless, the certainty of evidence is limited by the heterogeneity across studies in terms of inclusion criteria, study design, intervention duration and specific formulas/thickeners evaluated, and by methodological concerns, particularly unblinded outcome assessment and reliance on caregiver-reported symptoms. Among the thickening agents, locust bean gum, cornstarch, and rice starch were most commonly used, with some trials showing favorable outcomes in symptom reduction. Currently, there is no robust evidence to support the preferential use of one thickening agent over another, owing to the paucity of comparative studies. Overall, AR formulas are considered superior to the practice of adding thickeners to standard formulas, as AR formulas exhibit a standardized and homogeneous composition, with osmolarity and caloric density closely matching that of standard formulas [59].

Extensively or partially hydrolyzed protein [26, 31, 48, 49, 51], and soy-based [43] formulas also showed some benefit. However, heterogeneity in study design and outcome measures made direct comparisons difficult, and evidence that protein hydrolyzation offers additional benefit to formula thickening is lacking. The certainty of evidence tends to be very low for most RCTs, due to very serious risk of bias and small sample size. As for maternal milk fortification, two trials were included [25, 27], showing conflicting effects on GER. Therefore, no conclusions can be drawn based on the available evidence.

Our results are in line with the ESPGHAN Position Paper on the use of infant formulas for the treatment of functional gastrointestinal disorders [12] that recommends to consider thickened feeds only in selected cases of significant regurgitation, while confirming the lack of evidence to support the use of hydrolyzed proteins.

### Probiotics

We identified only small RCTs on probiotics supplementation [21, 31, 32], mainly *Lactobacillus reuteri* and *Bifidobacterium animalis*, and one systematic review with meta-analysis including 6 studies [28], with mixed results. The pathophysiology of functional regurgitation is multifactorial, and a role of early life events [60].

The effect of probiotics on upper gastrointestinal motility has been hypothesized to be their ability to modulate intestinal inflammation and improve gut motility [61]. Even though our data show that probiotic supplementation seems associated with an improvement in GER frequency or I-GERQ-R score, in the absence of serious adverse effects, the variability in type of probiotic administered, duration of supplementation and outcomes assessed makes the results non-generalizable. Of note, the included trials generally had low risk of bias in outcome assessment due to the use of placebo controls and objective endpoints, such as weight gain.

### Positioning therapy

Seven RCTs [8, 34, 40–42, 46] reporting data on several infant positions were included in the review. Most of the studies reported improvements in reflux episodes with left lateral or head-elevated positions; however, these studies commonly exhibited high risk of bias due to lack of blinding and use of subjective outcomes (e.g., caregiver-reported symptoms). In addition, since one of the main risk factors for SIDS is represented by prone or side sleep position [62] and that supine position on a flat, non-inclined surface is recommended for every sleep, even for infants with GER [63], the clinical applicability of alternative positions is limited.

### Alginates

Four trials on alginates were included in the review [55–58]. Alginates mechanism of action is related to their ability to transform into a foam floating atop the gastric contents, after contact with gastric acid. During episodes of GER, alginates ascend into the esophagus ahead of acidic gastric contents and may also function as a physical barrier, thereby reducing the frequency of reflux events [64]. Overall, alginates appeared to reduce symptoms in several studies using placebo comparators, supporting their potential role as a non-pharmacologic option for infants with more severe symptoms [56, 58]. Nevertheless, variations in product formulations and outcome measures, as well as concerns about blinding and allocation concealment, reduced the overall certainty of the evidence.

### Manual therapies

Manual therapies, such as massage or abdominal techniques, were explored in only two studies, yielding preliminary but inconclusive results [33, 39]. These interventions may offer promise, particularly in integrative care models, but currently lack a robust evidence base.

Overall, outcome reporting was variable and often relied on subjective measures such as parental diaries or the I-GERQ- [65]. Objective measures (e.g., pH monitoring, weight gain) were used less frequently but generally associated with lower risk of bias. Importantly, adverse events were rarely reported, and no serious harms were identified, suggesting an acceptable safety profile for most non-pharmacological interventions.

This review highlights several limitations in the current evidence base: small sample sizes in many trials, limited follow-up durations, heterogeneity in intervention protocols and outcome definitions, and frequent methodological shortcomings, particularly in blinding and outcome assessment. Moreover, the overall certainty of evidence, assessed using the GRADE approach, was low to moderate for most interventions and outcomes.

Clinical implications of these findings suggest that while certain non-pharmacological strategies—such as thickened formulas and possibly alginates—may offer symptom relief for infants with GER, their use should be guided by individual patient needs, parental preferences, and safety considerations. Future research should prioritize high-quality, adequately powered RCTs using standardized outcome measures and longer-term follow-up, particularly in underrepresented populations such as preterm infants and older children.

## Conclusion

This systematic review provides an updated synthesis of the evidence on non-pharmacological interventions for the management of GER and GERD in infants and children. Among the evaluated strategies, thickened formulas and alginates showed the most consistent evidence of symptom improvement, though overall certainty remains limited by methodological concerns. For this reason, parental reassurance should always represent the first intervention for the management of infant regurgitation, while AR-formulas and alginates should be considered only in case of great parental distress.

Other interventions, such as probiotics, infant positioning, and manual therapies, demonstrated variable results and require further investigation. While non-pharmacological treatments appear generally safe, the current evidence is characterized by heterogeneity, small sample sizes, and frequent risk of bias. High-quality, well-controlled trials using standardized outcomes are needed to better inform clinical decision-making and optimize care for pediatric patients with GER and GERD.

## Supplementary Information

Below is the link to the electronic supplementary material.

Supplementary Material 1

## Data Availability

Materials about this study can be obtained from the corresponding author on reasonable request.

## Abbreviations

AR: Anti-regurgitation
AA: Antacid
ANCOVA: Analysis of Covariance
CFU: Colony-Forming Units
ESPGHAN: European Society for Pediatric Gastroenterology, Hepatology, and Nutrition
GER: Gastroesophageal Reflux
GERD: Gastroesophageal Reflux Disease
GRADE: Grading of Recommendations Assessment, Development and Evaluation
GSQ-I: Gastroesophageal Symptom Questionnaire for Infants
HE: Head-of-Cot Elevation
I-GERQ-R: Infant Gastroesophageal Reflux Questionnaire Revised
LLP: Left Lateral Position
MeSH: Medical Subject Headings
MII: Multichannel Intraluminal Impedance
NASPGHAN: North American Society for Pediatric Gastroenterology, Hepatology, and Nutrition
PICO: Population, Intervention, Comparator, Outcome
PPIs: Proton Pump Inhibitors
PRISMA: Preferred Reporting Items for Systematic Reviews and Meta-Analyses
PROSPERO: International Prospective Register of Systematic Reviews
RCT: Randomized Controlled Trial
RoB 2: Risk of Bias 2 Tool
SD: Standard Deviation
SIDS: Sudden Infant Death Syndrome
