# Supplementary material for: Efficacy and safety of non-pharmacological interventions for gastroesophageal reflux and gastroesophageal reflux disease in children: a systematic review
Source: Ital J Pediatr. 2026 May 7;52:108. doi: 10.1186/s13052-026-02264-z (PMC13322008; doi:10.1186/s13052-026-02264-z)
Supplement: Supplementary file 1 — Supplementary Material 1 [file 13052_2026_2264_MOESM1_ESM.docx]

### Additional File 1.

## Eligibility criteria

### Population

We included studies involving infants, children, and adolescents (0–18 years) with a confirmed diagnosis of GER or GERD, based on any recognized clinical or diagnostic criteria.

### Intervention

Non-pharmacological treatments for GER or GERD, including dietary modifications, feeding interventions, positional therapy, behavioral therapy, alginates, massage therapy, and complementary therapies.

### Comparator

Other non-pharmacological treatments, placebo or no treatment.

### Outcome

Following the GRADE approach, clinical experts identified the following as critical outcomes:

- Frequency of vomiting/regurgitation
- Infant Gastro-Esophageal Reflux Questionnaire Revised (I-GERQ-R)
- Total number of reflux events
- Estimated volume regurgitated
- Frequency of respiratory symptoms, nocturnal cough, asthma
- Weight gain
- Adverse events

### Study design

We included systematic reviews/meta-analyses of randomized controlled trials (RCTs) and individual RCTs.

## Information sources

Systematic literature searches were conducted in PubMed, Embase, and Web of Science on May 12, 2024. Filters were applied to identify English-language RCTs, systematic reviews, and meta-analyses. No publication date restrictions were applied. We also screened the reference lists of relevant articles to identify additional eligible studies.

## Search strategy

Search strategies were developed using both medical subject headings (MeSH) and free-text terms relevant to the PICO question. The complete search strategies for all three databases are provided in Additional File 1.

## Selection process

Two reviewers (MO and LG) independently screened all titles and abstracts retrieved from the databases using EndNote™ X7.4. Duplicates were automatically removed. Articles meeting the inclusion criteria were subsequently assessed in full text. Disagreements were resolved through discussion. Included studies are described in the main text and summary tables, while excluded studies, along with reasons for exclusion, are listed in Additional File 2.

## Data collection process

Data extraction was conducted by one reviewer and independently verified by another. Specifically, LG extracted data from the first half of the included studies, while MO extracted from the second half. Each reviewer then cross-checked the other’s work. Discrepancies were resolved by discussion. Data were collected using a standardized Microsoft Excel sheet.

## Data items

We extracted the following data:

- Study ID (first author, year of publication)
- Country of study
- Study design
- Population characteristics (condition, age, sex)
- Sample size
- Description of the intervention and comparator
- Main efficacy outcomes
- Main safety outcomes

## Risk of bias assessment

Risk of bias was assessed by one reviewer and verified by another, following the same procedure as for data extraction. RCTs were evaluated using the Cochrane RoB 2 tool, while systematic reviews were assessed using the JBI Critical Appraisal Checklist for Systematic Reviews and Research Syntheses.

## Statistical analysis

We did not perform meta-analyses due to the limited number of studies available for each comparison and the heterogeneity in PICO elements. Outcome results, as reported in the original studies, were presented in summary tables and GRADE evidence profiles. Continuous outcomes were expressed as means and standard deviations or standard errors, or as medians with interquartile ranges. Categorical outcomes were reported as frequencies and percentages. Reported p-values were retained from the original studies, and a p-value of <0.05 was considered statistically significant.

## Reporting bias assessment

We assessed outcome reporting bias within each study under domain 5 of the RoB 2 tool, focusing on selective reporting. When available, we compared trial protocols with final publications. In the absence of registered protocols, we compared outcomes described in the methods section with those reported in the results. Due to the limited number of studies per comparison, we did not assess the risk of publication bias.

## Certainty assessment

The certainty of evidence for outcomes rated as critical by clinical experts was assessed using the GRADE approach. Each outcome was graded as high, moderate, low, or very low certainty. Evidence profile tables were generated using GRADEpro GDT software.

### Literature search strategies

| PubMed, 12/05/2024 | | |
| --- | --- | --- |
| Search | Query | Results |
| #1 | gastroesophageal reflux OR gastro esophageal reflux OR gastrooesophageal reflux OR gastro oesophageal reflux OR GER OR GERD OR GOR OR GORD OR (gastric[Title/Abstract] AND (acid[Title/Abstract] OR reflux[Title/Abstract])) OR (reflux[Title/Abstract] AND (oesophagitis[Title/Abstract] OR esophagitis[Title/Abstract])) OR (erosive[Title/Abstract] AND (oesophag*[Title/Abstract] OR esophag*[Title/Abstract])) OR (regurgitation[Title/Abstract] NOT (aortic[Title/Abstract] OR mitral[Title/Abstract] OR tricuspid[Title/Abstract] OR valve[Title/Abstract] OR valvular[Title/Abstract] OR paravalvular[Title/Abstract] OR pulmonary [Title/Abstract])) OR "acid reflux"[Title/Abstract] | 95,575 |
| #2 | child OR children OR pediatric OR pediatrics OR paediatric OR paediatrics OR infant OR infants OR infancy OR newborn OR newborns OR adolescent OR adolescents OR juvenile OR youth OR toddler OR toddlers OR kid OR kids OR boy OR boys OR girl OR girls OR baby OR babies OR teen OR teens OR preteen OR preteens OR teenager OR teenagers OR pubescen* OR prepubescen* OR neonate OR neonates OR (allchild[Filter] OR newborn[Filter] OR allinfant[Filter] OR infant[Filter] OR child[Filter] OR adolescent[Filter] OR preschoolchild[Filter]) | 6,805,330 |
| #3 | "Complementary Therapies"[Mesh] OR "Food"[Mesh] OR "Food Additives"[Mesh] OR "food, formulated"[Mesh] OR "infant formula"[Mesh] OR "Milk"[Mesh] OR "Diet"[Mesh] OR "Posture"[Mesh] OR "dietary supplements"[Mesh] OR "Life Style"[Mesh] OR "weight loss"[Mesh] | 1,421,818 |
| #4 | (non-pharmacologic*[Title/Abstract] OR nonpharmacologic*[Title/Abstract] OR position*[Title/Abstract] OR postur*[Title/Abstract] OR alcohol[Title/Abstract] OR life style*[Title/Abstract] OR lifestyle*[Title/Abstract] OR diet*[Title/Abstract] OR milk*[Title/Abstract] OR soy[Title/Abstract] OR soya[Title/Abstract] OR homeopath*[Title/Abstract] OR massag*[Title/Abstract] OR oil[Title/Abstract] OR oils[Title/Abstract] OR acupunctur*[Title/Abstract] OR hypnother*[Title/Abstract]) | 2,270,978 |
| #5 | (parent*[Title/Abstract] AND (guid*[Title/Abstract] OR support*[Title/Abstract] OR educ*[Title/Abstract] OR teaching[Title/Abstract])) | 136,319 |
| #6 | ((complementary[Title/Abstract] OR alternative[Title/Abstract]) AND (therap*[Title/Abstract] OR treatment*[Title/Abstract] OR option*[Title/Abstract] OR intervention*[Title/Abstract] OR medicin*[Title/Abstract])) | 344,312 |
| #7 | ((food[Title/Abstract] OR feed*[Title/Abstract]) AND (modification*[Title/Abstract] OR advice*[Title/Abstract] OR intervention*[Title/Abstract] OR thicke*[Title/Abstract])) | 102,873 |
| #8 | "probiotics"[Mesh] OR "prebiotics"[Mesh] OR (probiotic*[Title/Abstract] OR prebiotic*[Title/Abstract] OR pro-biotic*[Title/Abstract] OR pre-biotic*[Title/Abstract]) | 56,205 |
| #9 | #3 OR #4 OR #5 OR #6 OR #7 OR #8 | 3,580,341 |
| #10 | #1 AND #2 AND #9 | 4,092 |
| #11 | #10 AND ((clinicalstudy[Filter] OR clinicaltrial[Filter] OR comparativestudy[Filter] OR controlledclinicaltrial[Filter] OR meta-analysis[Filter] OR randomizedcontrolledtrial[Filter] OR systematicreview[Filter]) AND english[Filter]) | 803 |

| Embase, 12/05/2024 | | |
| --- | --- | --- |
| Search | Query | Results |
| #1 | 'gastroesophageal reflux'/exp OR 'gastroesophageal reflux':ti,ab,kw OR 'gastro esophageal reflux':ti,ab,kw OR 'gastrooesophageal reflux':ti,ab,kw OR 'gastro oesophageal reflux':ti,ab,kw OR ger:ti,ab,kw OR gerd:ti,ab,kw OR gor:ti,ab,kw OR gord:ti,ab,kw OR (gastric:ti,ab,kw AND (acid:ti,ab,kw OR reflux:ti,ab,kw)) OR (reflux:ti,ab,kw AND (oesophagitis:ti,ab,kw OR esophagitis:ti,ab,kw)) OR (erosive:ti,ab,kw AND (oesophag*:ti,ab,kw OR esophag*:ti,ab,kw)) OR (regurgitation:ti,ab,kw NOT (aortic:ti,ab,kw OR mitral:ti,ab,kw OR tricuspid:ti,ab,kw OR valve:ti,ab,kw OR valvular:ti,ab,kw OR paravalvular:ti,ab,kw OR pulmonary:ti,ab,kw)) OR 'acid reflux':ti,ab,kw | 143,723 |
| #2 | child:ti,ab,kw OR children:ti,ab,kw OR pediatric:ti,ab,kw OR pediatrics:ti,ab,kw OR paediatric:ti,ab,kw OR paediatrics:ti,ab,kw OR infant:ti,ab,kw OR infants:ti,ab,kw OR infancy:ti,ab,kw OR newborn:ti,ab,kw OR newborns:ti,ab,kw OR adolescent:ti,ab,kw OR adolescents:ti,ab,kw OR juvenile:ti,ab,kw OR youth:ti,ab,kw OR toddler:ti,ab,kw OR toddlers:ti,ab,kw OR kid:ti,ab,kw OR kids:ti,ab,kw OR boy:ti,ab,kw OR boys:ti,ab,kw OR girl:ti,ab,kw OR girls:ti,ab,kw OR baby:ti,ab,kw OR babies:ti,ab,kw OR teen:ti,ab,kw OR teens:ti,ab,kw OR preteen:ti,ab,kw OR preteens:ti,ab,kw OR teenager:ti,ab,kw OR teenagers:ti,ab,kw OR pubescen*:ti,ab,kw OR prepubescen*:ti,ab,kw OR neonate:ti,ab,kw OR neonates:ti,ab,kw | 3,532,866 |
| #3 | 'alternative medicine'/exp OR 'food'/exp OR 'food additive'/exp OR 'elemental diet'/exp OR 'artificial milk'/exp OR 'milk'/exp OR 'diet'/exp OR 'body position'/exp OR 'dietary supplement'/exp OR 'lifestyle'/exp OR 'body weight loss'/exp | 2,283,556 |
| #4 | (non-pharmacologic*:ti,ab,kw OR nonpharmacologic*:ti,ab,kw OR position*:ti,ab,kw OR postur*:ti,ab,kw OR alcohol:ti,ab,kw OR life style*:ti,ab,kw OR lifestyle*:ti,ab,kw OR diet*:ti,ab,kw OR milk*:ti,ab,kw OR soy:ti,ab,kw OR soya:ti,ab,kw OR homeopath*:ti,ab,kw OR massag*:ti,ab,kw OR oil:ti,ab,kw OR oils:ti,ab,kw OR acupunctur*:ti,ab,kw OR hypnother*:ti,ab,kw) | 1,546,146 |
| #5 | (parent*:ti,ab,kw AND (guid*:ti,ab,kw OR support*:ti,ab,kw OR educ*:ti,ab,kw OR teaching:ti,ab,kw)) | 184,834 |
| #6 | ((complementary:ti,ab,kw OR alternative:ti,ab,kw) AND (therap*:ti,ab,kw OR treatment*:ti,ab,kw OR option*:ti,ab,kw OR intervention*:ti,ab,kw OR medicin*:ti,ab,kw)) | 498,345 |
| #7 | ((food:ti,ab,kw OR feed*:ti,ab,kw) AND (modification*:ti,ab,kw OR advice*:ti,ab,kw OR intervention*:ti,ab,kw OR thicke*:ti,ab,kw)) | 142,264 |
| #8 | 'probiotic agent'/exp OR 'prebiotic agent'/exp OR (probiotic*:ti,ab,kw OR prebiotic*:ti,ab,kw OR pro-biotic*:ti,ab,kw OR pre-biotic*:ti,ab,kw) | 78,864 |
| #9 | #3 OR #4 OR #5 OR #6 OR #7 OR #8 | 3,713,840 |
| #10 | #1 AND #2 AND #9 | 4,521 |
| #11 | #10 AND ([article]/lim OR [article in press]/lim OR [review]/lim) AND [english]/lim AND ('clinical trial'/de OR 'clinical trial topic'/de OR 'comparative effectiveness'/de OR 'controlled clinical trial'/de OR 'controlled study'/de OR 'double blind procedure'/de OR 'meta analysis'/de OR 'meta analysis topic'/de OR 'randomized controlled trial'/de OR 'randomized controlled trial topic'/de OR 'systematic review'/de OR 'systematic review topic'/de) | 868 |

| Web of Science, 12/05/2024 | | |
| --- | --- | --- |
| Search | Query | Results |
| #1 | TS=(gastroesophageal reflux OR gastro esophageal reflux OR gastrooesophageal reflux OR gastro oesophageal reflux OR GER OR GERD OR GOR OR GORD OR (gastric AND (acid OR reflux)) OR (reflux AND (oesophagitis OR esophagitis)) OR (erosive AND (oesophag* OR esophag*)) OR (regurgitation NOT (aortic OR mitral OR tricuspid OR valve OR valvular OR paravalvular OR pulmonary )) OR "acid reflux") | 97,816 |
| #2 | TS=(child OR children OR pediatric OR pediatrics OR paediatric OR paediatrics OR infant OR infants OR infancy OR newborn OR newborns OR adolescent OR adolescents OR juvenile OR youth OR toddler OR toddlers OR kid OR kids OR boy OR boys OR girl OR girls OR baby OR babies OR teen OR teens OR preteen OR preteens OR teenager OR teenagers OR pubescen* OR prepubescen* OR neonate OR neonates) | 3,331,552 |
| #3 | TS=(complementary therap* OR food OR infant formula OR milk OR diet OR posture OR dietary supplement* OR life style OR lifestyle OR weight loss) | 2,369,021 |
| #4 | TS=((non-pharmacologic* OR nonpharmacologic* OR position* OR postur* OR alcohol OR life style* OR lifestyle* OR diet* OR milk* OR soy OR soya OR homeopath* OR massag* OR oil OR oils OR acupunctur* OR hypnother*)) | 4,530,943 |
| #5 | TS=((parent* AND (guid* OR support* OR educ* OR teaching))) | 206,073 |
| #6 | TS=(((complementary OR alternative) AND (therap* OR treatment* OR option* OR intervention* OR medicin*))) | 461,633 |
| #7 | TS=(((food OR feed*) AND (modification* OR advice* OR intervention* OR thicke*))) | 157,677 |
| #8 | TS=("probiotics" OR "prebiotics" OR (probiotic* OR prebiotic* OR pro-biotic* OR pre-biotic*)) | 77,645 |
| #9 | #3 OR #4 OR #5 OR #6 OR #7 OR #8 | 6,199,990 |
| #10 | #1 AND #2 AND #9 | 3,228 |
| #11 | #10 AND Article or Review Article (Document Types) AND English (Languages) AND TS=(systematic review OR meta-analysis OR randomi* control* trial OR RCT OR clinical trial OR comparative) | 381 |
